# Impact of Legal Traditions on Forensic Mental Health Treatment Worldwide

**DOI:** 10.3389/fpsyt.2022.876619

**Published:** 2022-04-25

**Authors:** Pavlos Beis, Marc Graf, Henning Hachtel

**Affiliations:** Department of Forensics, University Psychiatric Clinic Basel, Basel, Switzerland

**Keywords:** forensic psychiatry, mental health, forensic service, legal frameworks, treatment standards

## Abstract

**Background:**

Forensic psychiatry is a subspecialty dealing with the diagnosis and treatment of mentally ill offenders. However, forensic treatment standards vary. Differences arise among forensic treatment standards, due to variations in either the legal framework, the general psychiatric treatment standards, or the forensic training standards. Thus, to date there is no evidence-based pattern for how forensic services should be organized and provided.

**Aims:**

The aim of this article is to compare forensic services in various countries in order to contribute to the current debate on international forensic treatment standards, by informing about existing differences in available policies.

**Methods:**

This scoping review was conducted by reviewing the academic literature regarding forensic treatment around the world. Studies were identified from Pub-Med and Google-Scholar. Keywords for the search included “forensic psychiatry,” “mentally ill offenders,” “legal framework,” “jurisdiction,” and the names of geographical regions.

**Results:**

Forensic treatment admission varies significantly around the world. There are countries that do not recognize forensic psychiatry as a subspecialty, whereas other countries apply insufficient forensic training. Most countries provide inpatient treatment for mentally ill offenders. However, service organization varies, including where the services are delivered (prisons, high-security hospitals, and general psychiatric departments). Forensic services are mainly centralized, although the need for outpatient care is emerging. This manuscript updates the findings of a chapter by Anne G. Crocker, James D. Livingston, and Marichelle C. Leclair that conducted an international review on the organization of forensic mental health services internationally, by legal framework. We were also inspired by the classification of legal frameworks from that chapter conducting the present review. Building upon that chapter we reviewed current literature about forensic mental health treatment from countries with different legal traditions, accentuated similarities and differences among them and highlighted that further follow-up research is needed, aiming the optimization of forensic treatment standards.

**Discussion:**

Differences may originate mainly from variations in the legal tradition. These differences combined with the limited evidence on the effectiveness of the intervention imply the need for the optimization of forensic treatment standards on an international level. Therefore, further follow-up studies are needed.

## Introduction

Forensic psychiatry is a special field of psychiatry, which deals with subject-specific assessment questions and the treatment of mentally ill lawbreakers (1). In recent years, there has been a noteworthy increase in demand for forensic psychiatric services worldwide (2), with various factors contributing to this phenomenon; deinstitutionalization since the 1970's has often resulted in an increasing number of mental ill individuals living in the community, an intended outcome. However, an unintended outcome has been that the dysfunctional behavior of some deinstitutionalized people has often come to the attention of the police, who have been more likely to address criminal charges (3). Public intolerance of non-conforming behavior and the elevated media reporting of violence may also have contributed to this phenomenon (4). However, forensic mental services are defined and provided differently across the world (5), although the underlying clinical issues are similar throughout the different countries: a fact that could be explained by differences in legal frameworks and, to a questionable degree, cultural differences (2, 36).

In order to understand how the different forensic mental health systems are organized, it is essential to examine the legal frameworks which determine who will receive these services and how they will be offered. The four main legal frameworks are: common law, civil law, Islamic law (shari'ah) and the legislation of former communist countries (6) (Table 1).

**Table 1:** Fitness to plead, mental disorder defense, diminished responsibility, and discharge provisions by legal framework (6).

**Legal framework**	**Fitness to plead**	**Mental disorder defense**	**Diminished responsibility**	**Discharge provisions**
Common Law	Provided by most countries	Provided by most countries	Not common for offenses other than homicide	Varies from ministerial assent (e.g., Australia) to courts or treating psychiatrists
Civil Law	Mostly not provided (notable exception: Germanic Legal family)	Provided by most countries	Provided by most countries	Mainly responsibility of the court
Islamic Law	n/a^*^	Provided by most countries, although information is scarce	n/a^*^	n/a^*^
Legislation of former communist countries	Provided by most countries	Provided by most countries	Provided by most countries	Mainly responsibility of the court

* *Not available*.

*Table 2 Forensic service and total number of psychiatric beds by country*.

### Legal Frameworks

#### Common Law

The common law legal tradition is practiced in all countries whose legal system developed from the Anglo-Saxon and it derives from the informal way justice was applied in the Anglo-Saxon kingdoms, especially in the practices of the courts of the English kings in the years that followed the Norman Quest of 1066. It is considered to have a pragmatic approach and to be less prescriptive in nature compared with the civil law legal tradition. Common law is characterized by the adversarial approach, where the “truth” is believed to come from the “*choc des opinions”* (battle of opinions). The rule of the court is more passive and the judge serves as an arbiter that leaves the presentation to the parties. In contrast to the civil law, there is no concept of responsibility regarding the psychological element (50, 51). Most countries that adopt the common-law legal system, provide procedures for evaluating the fitness of an accused to stand trial. However, there are significant differences regarding the duration of detention for individuals found unfit to plead. For example, in Canada there is no time limit to the detention in hospital of an unfit accused, but in New Zealand permanently unfit individuals must be brought before the court after serving half of their sentence. The mental disorder or insanity defense is also available in most common-law countries (6). On the other hand, the availability of diminished responsibility for offenses other than homicide is not common in the common-law legal systems (8).

#### Civil Law

The civil law legal tradition, sometimes referred to as Roman-Germanic law, originates from the ancient Roman and Greco-Roman tradition. The current legislation reflects the major changes it experienced during the Middle Ages, the Holy Roman Empire and the French Revolution. The criminal process begun not with an accusation but with a suspicion and the authorities had to prove a case against the citizen. Thus, civil law originates from a public policy relating to the rise of power of the government, was adjusted however after the Enlightment and the French Revolution. It is characterized by the inquisitorial approach, where the “truth” is believed to come through extensive investigation and examination of all evidence (51). The judge has a much more active role, in comparison to the adversarial system, as the court is responsible not only for the right decision but also for the investigation that leads to the decision. In the civil law system, penal codes declare what is an offense and what is not and stipulate procedures, which must be applied by the magistrates with little discretionary power. Regarding the mentally disorder offenders, the civil law tends to emphasize the psychological element, as responsibility is the basic concept (50). Most countries that adopt the civil-law legal systemdo not assess the fitness to plead of mentally ill offenders (6). There are however exceptions, for example Germany, which uses criteria similar to the common-law jurisdictions to determine fitness to stand trial (9). In addition, most civil-law countries provide mental disorder defense to various degrees. For example, personality disorders are excluded from the mental disorder defense in France, whereas nearly 37% of the patients in forensic psychiatric hospitals in Germany have a primary diagnosis of personality disorder (10). Finally, diminished responsibility is available in almost all civil-law countries and typically results in a diminished sentence (8).

#### Islamic Law (Shari'ah)

Shari'ah is an Arabic word meaning “pathway to be followed.” Shari'ah is applied in various countries of the Middle-East, Africa and Asia, with Saudi Arabia applying its purest form. The bibliography in English regarding the Islamic Law and it's reflection upon mental health is limited. The concept of criminal responsibility is generally accepted in the Islamic-law countries. Insanity in Shari'ah is classified as either continuous, intermittent or partial. Involuntary admission to a psychiatric hospital is possible in the Islamic-law countries, considering however only the need for therapy and not the dangerousness of the subject (11).

#### Legislation of Former Communist Countries (Socialist Law)

Socialist law designates the legal tradition which has been used in communist and ex-communist countries. There is debate about whether socialist law constitutes a separate legal tradition. It derives from the civil-law legal tradition but has major differences in accordance with the Marxist-Leninist ideology, mainly regarding public and private law. During the Cold War, the majority of the comparative law theorists considered the socialist law as a separate legal system, focusing on the legal consequences of a system grounded in Marxist materialist ideology mandating public ownership over the means of production (52). Several features have been identified as distinguishing socialist law from civil law: that socialist law was programmed to die out with the disappearance of private property, that a single political party dominated in socialist countries, that law was subordinated to the creation of a new economic order as private law was absorbed by public law, that law had a religious character and that law was prerogative instead of normative (53). However, since 1990 radical changes have been implemented in the former-communist states, a fact which has been descripted as a “return” to the civil law legislation. There is however an ongoing debate, whether judicial and administrative structures instituted during the communist era continued to alter the forensic assessment (54). The evaluation of fitness to stand trial is mentioned in many former communist countries. In addition, criminal irresponsibility owing to a mental disorder exists in the majority of the former communist countries, but this will however be determined differently amongst them, regarding inpatient or outpatient compulsory treatment (12).

## Methods

Due to the complex, large and heterogeneous nature of the available information on the forensic legal systems and their impact on forensic treatment and due to the fact, that forensic legal systems are not expected to rapidly change over time a scoping review methodology was followed. This scoping review included five steps: (1) identifying the research question; (2) identifying relevant studies; (3) study selection; (4) charting the data; and (5) summarizing and reporting the results.

### Research Question

The review was guided by the question: *What is the impact of the different legal traditions on forensic mental health treatment around the world and how do these differences alter the forensic treatment?*

### Research Strategy

An academic literature search regarding forensic mental health treatment standards was conducted using PubMed and Google Scholar with a timeframe from 2010 to 2021. Specifically, articles of potential interest were identified by using the following terms: “forensic psychiatry,” “legal framework,” “mentally ill offenders,” “jurisdiction,” and the names of the geographical continents (Europe, Africa, North America, South America, Asia, and Australia).

### Study Selection

The selected articles were included in the reference list only if meeting the eligibility criteria, which included publications (I) available in English and German, and providing information about (II) the presence of forensic assessments and services, (III) the integration of forensic services into the general mental health services, (IV) the specific forensic psychiatric treatment standards, or (V) post-discharge services. Articles not written in English or German, those evocating forensic treatment as a secondary subject and those focusing primarily on ethical questions, or repeating previous authors were excluded.

### Screening for Relevance

**Figure 1:**
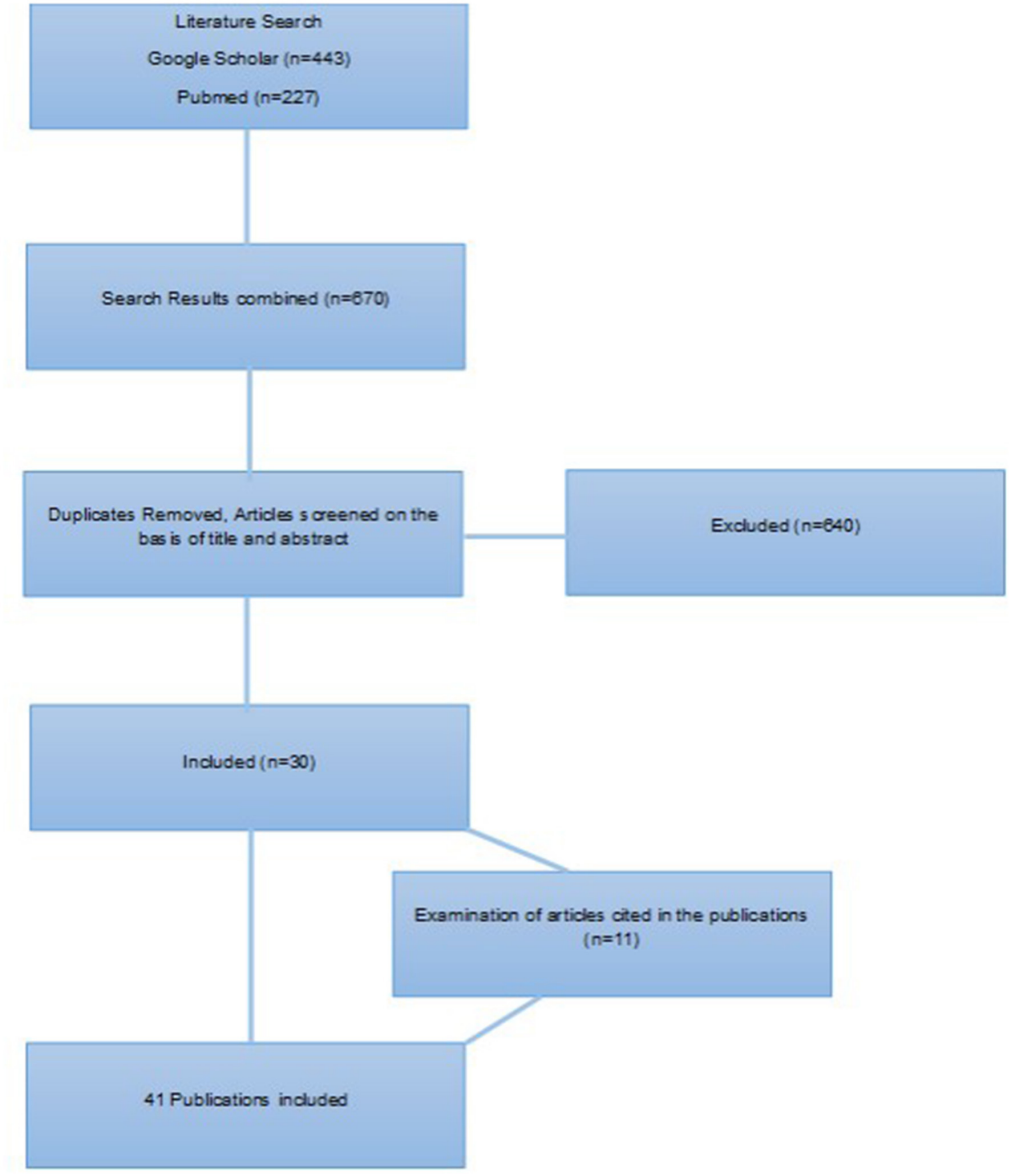
Flowchart of study selection.

The research identified a total of 443 articles in Google Scholar and 227 articles in PubMed. After duplicates were removed, the titles and abstracts were screened manually. Some additional references identified by manual search in the reference list from the retrieved articles were included, with the condition that they met the inclusion criteria. One older publication was found to be exceptionally helpful, thus it was included (Figure 1).

### Reporting the Results

A total of 38 publications, two national reports and one book chapter were included. Subsequently, the publications were divided per geographical continent (Europe: 14, Americas: 6, Asia: 11, Africa: 6, and Oceania: 4). For the purpose of conducting the introduction and the statistical data (Table 2), that according to our opinion help understanding the information reported in the results, additional literature was manually searched and is citied, thus in the end 70 publications were citied.

**Table 2:** Forensic service and total number of psychiatric beds in countries mentioned in the results.

**Countries**	**Location of forensic services**	**Forensic beds**	**Total psychiatric care beds**
**Europe**			
United Kingdom	Secure hospitals	England and Wales: 750 high secure, 3,500 medium secure (2014) (9) Scotland: 144 high secure, 146 medium secure (2021) (57)	22,475 (2020) (23)
France	Secure units in psychiatric hospitals, psychiatric hospitals	440 in UHSAs, 656 in UMDs (2014) (45)	54,991 (2019) (69)
Austria	Forensic hospital, forensic departments in psychiatric hospitals	384 (2005) (50)	6,099 (2019) (69)
Netherlands	Forensic Facilities (TBS-hospitals or forensic hospitals), forensic units in psychiatric hospitals	1,867 (2013) (9)	13,895 (2019) (69)
Finland	Psychiatric hospitals, wards	450 (2013) (55)	3,266 (2019) (69)
**Americas**			
Canada	Province-specific forensic units	1,523 (2006) (68)	13,714 (2019) (69)
United States	State-specific forensic units	7,835 (2015) (43)	81,799 (2018) (69)
Brazil	Forensic hospitals, psychiatric hospitals, prisons	3,677 (2015) (40)	25,097 (2016) (58)
Chile	Forensic units	209 (2012) (66)	2,703 (2020) (69)
**Asia**			
China	Ankang hospitals	7,000 (2012) (66)	506,637 (2018) (59)
Japan	Forensic Units	666 (2011) (56)	326,666 (2019) (69)
Russia	Secure hospitals, psychiatric hospitals	5,440 high secure, 6,582 medium secure (2014) (29)	155,834 (2008) (60)
Iran	Prisons, psyachiatric hospitals	n/a	6,716 (2006) (65)
Pakistan	Prisons, psyachiatric hospitals	33 (2009) (61)	3,100 (2009) (61)
**Africa**			
Egypt	Psychiatric hospitals	840 (2006) (64)	6,324 (2006) (64)
South Africa	Forensic units in psychiatric hospitals	1,676 (2007) (62)	11,688 (2007) (62)
Nigeria	Forensic units, prisons	22 (2006) (63)	1,248 (2006) (63)
**Oceania**			
Australia	State-specific forensic units	680 (2020) (67)	10,513 (2020) (67)
New Zealand	Forensic units	221 (2005) (34)	1,609 (2020) (69)

## Results

In comparison to other medical fields, mental health, and especially forensic psychiatry, are probably most affected by the law, a fact that strengthens the need for incorporation between psychiatry and the different legal approaches aiming at a specific human behavior (37). In contrast, forensic psychiatry is not recognized as a separate subspecialty by a number of countries and even among the countries that do recognize it, there are significant differences regarding the training of forensic professionals (38). Thus, standardization in the practice of forensic psychiatry remains a challenge. In addition forensic treatment capacity varies regarding the number of forensic beds as well as regarding the location of forensic services (see Table 2 for selection of forensic services in the countries mentioned in the results). It is important to note that available information about forensic treatment standards varies among different countries. Europe and North American countries provide an abundance of publications, whereas there is only limited information available from African, Asian and South American countries. Therefore, a detailed review of the forensic treatment standards was only partially possible for those regions. Some important differences between countries and cultures are listed below.

### Europe

Forensic psychiatry is thought to be bound to the national and jurisdictional limits of each country's services (13). However, the European continent has been an example of exchanging information and techniques, mainly due to the European integration process of the European Union or the need to establish international guidelines. Nonetheless, differences regarding the legal frameworks, demographics and psychiatric traditions complicate this effort (8). For example, in common-law countries, such as the UK and Ireland, penal procedures are adversarial from the beginning, whereas in civil-law countries such as France, a single judge has the mission to decide whether there is enough evidence to proceed with the trial [8, 14). Most European countries provide forensic services in specialized clinics or hospitals; some such as Belgium, the Czech Republic, Greece and Switzerland also provide treatment in prisons (8). More specifically, in the United Kingdom, forensic services are organized by the level of security and are not divided into forensic or non-forensic clinics or wards. There are three kinds of inpatient facilities: high, medium and low security. Currently there are three high-security hospitals and around 60 medium secure units, providing 700 and 3,500 beds, respectively (44).

In France, the mental health care system in correctional facilities is organized similarly to the community psychiatric system, in three levels (outpatient care unit, day-treatment hospital, and specially equipped clinic). Only 26 out of France's 188 prisons provide day-treatment hospitals (*services medico-psychologiques regionaux*) (45). Similar to the UK, mentally ill offenders and aggressive non-offenders may be treated in secure psychiatric hospitals (6, 8). In the nine UHSAs (*unites hospitalières spécialement aménagées*), that were established in the 2010's and offer a capacity of 440 beds, mentally ill prisoners can be hospitalized either with their consent or against their will. In contrast, general psychiatric incarcerated patients can only be hospitalized in these clinics involuntarily. Maximum security psychiatric clinics, or the UMDs (*unités pour maladies difficiles*), are designed for forensic and non-forensic patients who could pose a high risk of aggression. Court-ordered treatment was implemented in 1998 in France for sexual offenders and other serious non-sexual crimes. The judge decides the duration of this care (up to 10 years) and the *médecin coordonnateur (coordinating doctor)* is required to write annual reports about the progress of the treatment. In contrast, France lacks practical handbooks or guidelines on writing forensic reports (14).

The Austrian forensic provision is centralized, provided by a high-security hospital (available only for men) and various small forensic departments within general psychiatric hospitals (8). In the Netherlands, forensic services are federalized. TBS-patients (*Terbeschikking Stelling, literally meaning* “*making a person available for psychiatric treatment*”) may be treated in TBS hospitals or forensic clinics within general psychiatric hospitals (8). After a slow increase in the average length of stay in the TBS system, hospitals must now apply for escorted furlough for every TBS patient within a year, unescorted within 4 years and transmural furlough within 6 years. For patients who require further treatment there are specific long-stay services, from which, after a recent unfortunate event, patients cannot leave unescorted (9).

In Finland, forensic treatment is offered mainly by the forensic state mental hospitals, which provide ~450 beds. The average length of stay is 10 years; however, every involuntary hospitalization is reviewed every 6 months by an expert, independent from the treatment facility (46). Before final discharge, the patient must attend provisional outpatient treatment for a maximum of 6 months (46). After successful provisional discharge and with low risk of reoffending, the involuntary treatment is terminated (5). Another notable example is Italy that closed all forensic psychiatric hospitals in 2008, aiming for the deinstitutionalization of all psychiatric patients, according to an Act of the Presidency of the Council of Ministers (18). The Act ordered that every Region should have forensic psychiatric services for their catchment areas (70). This fostered the need for new structures, the so-called “REMS” (Residents for the Execution of Security Measures), community-based mental health facilities, which provide for the treatment and management of socially dangerous offenders (16). The process of discharging patients from the old forensic psychiatric hospitals lasted 22 months, from April 2015 to January 2017, when Italy became the first country worldwide to close all forensic psychiatric hospitals (70). The development of the “REMS” was inspired by the experience of the community mental health services, hence “REMS” emphasize on social community-oriented vocational and recreational activities. Thus, patients who are considered suitable are granted accompanied leaves (70). However, there is criticism about this new structure, mainly because mentally ill offenders must serve part of their sentence in prison (18), which, combined with the small number of forensic beds, leads to the creation of long waiting lists (17). In addition, the autonomy permitted to patients in managing their daily routine, which roots from the community-oriented character, could pose difficulties for more sever ill individuals, who need greater motivation to actively participate in the recovery process (70).

Moreover, there is a significant variation regarding the training of forensic psychiatrists, e.g., 6 years in Finland, 3 years in England, and 6 months in Portugal (8). In almost all European countries there has nevertheless been an increase in the number of mentally ill criminals removed from the criminal justice system. This has eventually led to an increase of forensic beds (10), accompanied by a decrease in the average length of stay, which has however remained remarkably different throughout the European countries (e.g., 10 times greater in the Netherlands as in Slovenia) (15). On the other hand, some countries such as Romania do not recognize forensic psychiatry as a subspecialty (12).

### Americas

A major variance in the American continent is the different legal systems: the North American countries (Canada and the United States) adopt the common-law system, whereas the Latin American countries adopt the civil-law juridical tradition (6). In Canada, the forensic mental health service is organized in a provincial and territorial model, thus leading to a significant variation in the forensic mental treatment standards throughout the country. However, all provinces incorporate high-security forensic facilities or forensic departments within psychiatric hospitals (7, 19). Forensic psychiatry was recognized as a subspecialty in psychiatry as late as September 2009 (39). Similar to Canada, in the Unites States forensic treatment provision varies from one state to another. The majority of the states provide special forensic departments, whereas the remaining states treat forensic patients in general psychiatric facilities. In recent years, there has been an increase in the number of programs supporting release and reintegration into society, due to the very large presence of people with mental disorders in prisons (43).

In South America, forensic services are significantly different: the lack of standardized mental health services, assessments and screening tools leads to an underestimated prevalence of psychiatric disorders among offenders or prisoners. In contrast to Canada or the United States, fitness to stand trial is not relevant and in some countries such as Brazil, juridical proceedings are not stopped even if the defendant is incompetent. An absence of mental health services is reported, in combination with a significantly low number of forensic beds; even Brazil, which is the country with the largest number of forensic beds in Latin America, is not capable of providing sufficient forensic services. Forensic mental treatment is mainly provided by general psychiatrists, as forensic psychiatry as a subspecialty is yet to be recognized in many South American countries (40). In Chile, there are currently one high-complexity forensic unit and three medium-complexity units for mentally ill offenders. There are also the so-called Defendant Assessment Units (*Unidad de Evaluacion de Personas Imputadas)*, which are units within the civil hospitals that treat offenders who are mentally ill but have not yet been convicted. When treatment in these facilities is not available, defendants are detained in prisons (47).

### Asia

China's mental health services differ significantly from western countries (21). The current legislative structure in China is similar to the common-law legal system, but has its own characteristics. A notable example is the obligation of the family members of the offenders, to keep them under strict surveillance and arrange their medical treatment. The diagnostic criteria used in forensic psychiatric assessment in China are included in the Chinese Classification and Diagnostic Criteria of Mental Disorders (CCMD), which are similar to the ICD-10 Criteria. Regarding forensic treatment, in China the “Ankang” hospitals (the name “Ankang” in Chinese means “peace and health”), one of the three groups of psychiatric hospitals, act as forensic hospitals, as their aim is to treat mentally ill patients who could pose a risk to the public or to themselves. However, these hospitals do not solely receive mentally ill offenders, nor is forensic psychiatric training required for psychiatrists who work in these hospitals, a fact that distinguishes the Ankang hospitals from forensic hospitals, as they are understood in the western world. Patients will be discharged for a period of time (“pretended discharge”), often 1 month, before a formal discharge takes place and they are often not followed up after discharge (20–22).

In Japan, the “Act on Medical Care and Treatment for Persons Who Have Caused Serious Cases under the Condition of Insanity,” enforced in 2005, altered the forensic services (24). Forensic patients are to be treated, when necessary, as inpatients for a period of ~18 months, which is divided into three stages: the acute phase, the recovery phase and the rehabilitation phase. Each phase aims for the stabilization of the patient, the acquisition of insight and the preparation of a supportive network after eventual discharge. When these conditions are fulfilled, the patient may receive outpatient treatment, generally lasting 3 years (23, 24).

In Russia, mentally disordered prisoners can be moved to a psychiatric hospital, if specific psychiatric care is needed, and they are transferred back to the prison as soon as they are considered stable. For patients who are not capable of completing their sentence, a special commission of psychiatrists can recommend release from the sentence and the compulsory hospitalization. There are three types of psychiatric hospitals: the general hospitals, the medium-secured and the high-secured hospitals. Forensic patients can be hospitalized along with non-offenders in all the different hospitals, depending on their status; however, high-secured hospitals receive only mentally ill offenders. After discharge, patients are normally followed up as outpatients (29).

India identifies some important distinctions. Offenders found not guilty by reasons of mental illness will be treated in prisons or less often in psychiatric hospitals, as not all of the ~50 state mental hospitals have forensic units (25, 26). The Mental Health Care Act introduced the shift from “custodial care” of mental hospitals to deinstitutionalization. Open wards in general psychiatric hospitals also offer treatment for mentally ill offenders (48). Interestingly, in India forensic psychiatry is involved in other social situations, for example marriage (26). According to the Hindu Act of 1955, the Special Marriage Act of 1954 and the Parsi Marriage Act of 1936, unsoundness of mind is a ground for a null and void marriage (25, 26); however intellectual disabilities are not clearly addressed (25).

The countries that adopt Islamic law also present a number of specific features. Currently there are more than 50 Islamic states, so it is difficult to describe the mental health provisions in all of them (11). In addition, the literature in English is limited. Some Islamic states such as Yemen, Saudi Arabia and the United Arab Emirates do not have specific legislation for mental health, while others possess outdated legal frameworks (11). In Iran, there are 10 forensic psychiatric departments whose duties are the performance of psychiatric examinations and the determination of mental competence. There is no secure mental hospital for forensic patients, who are mainly detained in prisons or general psychiatric hospitals (41). Forensic mental health provision in Iran, as in most Islamic states, is in accordance with the Islamic penal code, which is drawn from Islamic law. According to Islamic law, the therapeutic bond between the psychiatrist and the patient is sacred, which means that human justice cannot force a psychiatrist to reveal information. Also, according to Islamic law, only opinions from Muslim psychiatrists can be taken into consideration (11). In Pakistan, forensic psychiatry is practiced in accordance with the Pakistan Penal Code. Regularly prisons provide facilities for offenders who have been diagnosed with a mental illness. The majority of them spend <1 year in these facilities, while inpatient treatment rarely exceeds a period of 5 years. Interestingly enough, these forensic facilities do not have psychiatrists, but a postgraduate trainee under supervision visits the facility usually every 2 weeks. When necessary forensic patients can be transferred to a general psychiatric hospital (27). Also, according to various studies, the percentage of male forensic patients in the Arabic countries ranges between 86 and 100% (28).

### Africa

In Africa, forensic psychiatry has largely remained undeveloped, with regard to both the legal frameworks and the forensic services (31). Forensic units tend to be extensions of prisons (30). Many countries in North and West Africa lack trained personnel and provide outdated mental health legislation. Egypt, being the African country with the most up to date mental health legislation after the revision in April 2009, offers special forensic training. In most African countries, there are no forensic facilities, and mentally ill lawbreakers are treated either in prisons or in general psychiatric hospitals. South Africa, a notable exception, provides forensic services in seven specially designed hospitals (31). Also, mentally ill prisoners requiring ambulatory care may be referred to the nearest mental health unit (32). In Nigeria, prisons may provide mental health care to ill inmates. Healthcare is mainly provided from non-psychiatric personnel, meaning from nurses and allied staff in the prison clinic. Less often, mental healthcare is provided by a visiting psychiatrist. Medication may be funded either by the prison service or by the inmate's relatives (35). Moreover, the role of traditional perceptions in Africa is very well-established, so patients often seek help from traditional healers (30).

### Oceania

In Australia, each state has its own legal and forensic mental health system. However, all states operate secure hospitals, divided into high-security and medium-security hospitals (29). Discharged patients can be transferred to the community forensic mental health services, where a follow-up of 2–3 years takes place. Australia follows the example of other western countries, aiming for recovery-oriented care. For this purpose, recovery philosophy is being introduced. In the Victorian Institute for Forensic Mental Health (known as forensic care), the recovery philosophy was formally introduced in 2010, encouraging greater collaboration between clinicians and patients by creating patient working parties, educational groups and review meetings (33). In New Zealand, forensic services are also decentralized and entirely integrated into civil mental health services. All regions have medium-security services and outpatient services, aiming ultimately at the integration of outpatients into the general psychiatric service (34). Forensic treatment outcomes have been described as impressive, as follow-up studies showed that many discharged patients were either employed or living independently (42).

## Discussion

As this article indicates, there is a wide variation in forensic psychiatric services worldwide. As forensic psychiatry is influenced by the respective judicial, ethical and general psychiatric treatment standards of every country, it is very difficult, if not impossible, to compare the different forensic settings around the world. Nevertheless, some common conclusions can be observed. The vast majority of countries provide special inpatient care for mentally ill lawbreakers, either in forensic or general psychiatric hospitals. Furthermore, the need for specific forensic outpatient services is emerging, thus many countries have already or are trying to establish such treatment standards. On the contrary, a high prevalence of mentally ill individuals in prisons indicates that there is still a long road ahead. Intolerance of socially deviant behavior, shutting down of long-term care institutions for economic purposes, or inadequate preparation before discharge could be reasons for this phenomenon (10). Therefore, the necessity to improve forensic mental health standards remains on the agenda.

Another key aspect concerning forensic mental health services is the extent to which they are integrated into the general psychiatric services. A paradox can be observed: although community-based psychiatry has emerged in recent decades, forensic services in most countries remain highly centralized, and in less populous countries are often provided in a single forensic hospital. The duration of forensic hospitalization, mainly influenced by the legal frameworks, also varies, with some countries defining a minimum amount of time and others a maximum. The characteristics and demographics of forensic patients also vary in interesting ways; psychotic illnesses such as schizophrenia, along with affective disorders or organic mental disorders, mainly fulfill the forensic criteria, whereas patients with personality disorders represent a significant part of the forensic population only in some countries. Moreover, in specific states, forensic patients are almost all masculine, a fact which suggests a connection between the cultural place of women and the forensic assessment. Post-discharge forensic services also vary, from absence of follow-up treatment to fully organized outpatient wards. There is only limited information on follow-up studies, a fact that makes conclusions for the continuity or outcome of the services nearly impossible.

Forensic training is also different among the training programs. According to the European Psychiatric Association (EPA) guidance on forensic psychiatry, the evidence in forensic psychiatry is weak, thus quality trials are needed. Nevertheless, the available evidence suggests that forensic treatment can produce better outcomes than prison detainment alone (49). In addition, it is suggested that forensic organization which minimizes the length of stay is to be preferred. Risk assessment based on structured professional judgment is to be preferred, noting however the limitation of the tools. Early psychiatric treatment for mentally ill offenders is recommended, whereas community treatment cannot be recommended based on the available evidence. Clinicians should follow the general psychiatric guidance, in addition to that for offenders, giving heed to long-term detention and ethical issues (49).

Moreover, this review shows differences in the forensic context among different countries. These differences could also reflect, to a reasonable extent, cultural differences. In contrast to the general mental health literature, the place of culture in forensic psychiatry has not been widely researched. It has been noted, that allowing culture as a defense could undermine the fairness of the justice system, as inconsistent or arbitrary standards could be applied. However, cultural issues may arise regarding the appropriate use of fair and meaningful methods of neuropsychological testing or forensic assessment. In addition, framing behavior as influenced by culture could contribute to stereotyping and stigmatizing whole communities or groups. Nevertheless, recognition of the impact of culture might help in the determination of what interventions should be preferred in order to achieve rehabilitation (36).

In summary, the managing of mentally ill offenders is an indicator of a country's ability to maintain public safety and to preserve basic human rights (20). The differences that emerge from variations in the legal frameworks, combined with the limited available evidence and the fact that mentally disordered offenders have often a range of complex needs (49), imply the need for the optimization of forensic treatment standards on an international level. Compared with general medicine or psychiatry, forensic psychiatry is trailing regarding the development of evidence-based guidelines. Aiming that development, more systematic reviews on the effectiveness of the pharmacological treatment and psychotherapeutic approaches would be of greater importance. Given the variety of treatment standards, legal approaches and environmental influences, studies about the efficacy of these interventions might be complicated. However, further research on the follow-up care, and especially long-term follow-up studies on psychosocial outcomes, rehabilitation and reoffending, could bring helpful information regarding psychiatric and criminal recidivism. Finally, as this article indicates, specific cultural features may interfere, thus further research on the impact of culture in forensic psychiatry also seems to be required.

## Author Contributions

PB and HH wrote the initial article. MG revised the article critically for important intellectual content. All have given final approval of the current version.

## Conflict of Interest

The authors declare that the research was conducted in the absence of any commercial or financial relationships that could be construed as a potential conflict of interest.

## Publisher's Note

All claims expressed in this article are solely those of the authors and do not necessarily represent those of their affiliated organizations, or those of the publisher, the editors and the reviewers. Any product that may be evaluated in this article, or claim that may be made by its manufacturer, is not guaranteed or endorsed by the publisher.

## References

[1] Nedopil N. Forensische psychiatrie. Fortschr Neurol Psychiatr. (2007) 75:172–85. 10.1055/s-2006-932154 17354185

[2] Crocker AG Braithwaite E Côté G Nicholls TL Seto MC. To detain or to release? Correlates of dispositions for individuals declared not criminally responsible on account of mental disorder. Can J Psychiatry. (2011) 56:293–302. 10.1177/070674371105600508 21586195

[3] Jansman-Hart EM Seto MC Crocker AG Nicholls TL Côté G. International trends in demand for forensic mental health services. Int J Forensic Ment Health. (2011) 10:326–36. 10.1080/14999013.2011.625591

[4] Whitley R Berry S. Analyzing media representations of mental illness: lessons learnt from a national project. J Mental Health. (2013) 22:246–53. 10.3109/09638237.2012.745188 23323727

[5] Seppänen A Törmänen I Shaw C Kennedy H. Modern forensic psychiatric hospital design: clinical, legal and structural aspects. Int J Ment Health Syst. (2018) 12:58. 10.1186/s13033-018-0238-7 30377440 PMC6195744

[6] Crocker AG Livingston JD Leclair MC. Forensic mental health systems internationally. In: Roesch R Cook AN, editors, Handbook of Forensic Mental Health Services. New York, NY: Routledge (2017). p. 3–76

[7] Moran JE. Mental disorder and criminality in Canada. Int J Law Psychiatry. (2014) 37:109–16. 10.1016/j.ijlp.2013.09.010 24139078

[8] Salize HJ Lepping P Dressing H. How harmonized are we? Forensic mental health legislation and service provision in the European Union. Crimin Behav Mental Health. (2005) 15:143–7. 10.1002/cbm.6 16575818

[9] Edworthy R Sampson S Völlm B. Inpatient forensic-psychiatric care: legal frameworks and service provision in three European countries. Int J Law Psychiatry. (2016) 47:18–27. 10.1016/j.ijlp.2016.02.027 27055603

[10] Konrad N Lau S. Dealing with the mentally ill in the criminal justice system in Germany. Int J Law Psychiatry. (2010) 33:236–40. 10.1016/j.ijlp.2010.06.005 20667595

[11] Tzeferakos GA Douzenis AI. Islam, mental health and law: a general overview. Ann Gen Psychiatry. (2017) 16:28. 10.1186/s12991-017-0150-6 28694841 PMC5498891

[12] Tataru N Marinov P Douzenis A Novotni A Kecman B. Forensic psychiatry in some Balkan countries. Curr Opin Psychiatry. (2010) 23:472–80. 10.1097/YCO.0b013e32833cfc05 20683182

[13] Nedopil N Taylor P Gunn J. Forensic psychiatry in Europe: the perspective of the Ghent Group. Int J Psychiatry Clin Pract. (2015) 19:80–3. 10.3109/13651501.2014.967700 25263225

[14] Combalbert N Andronikof A Armand M Robin C Bazex H. Forensic mental health assessment in France: recommendations for quality improvement. Int J Law Psychiatry. (2014) 37:628–34. 10.1016/j.ijlp.2014.02.037 24631526

[15] Tomlin J Lega I Braun P Kennedy HG Herrando VT Barroso R. Forensic mental health in Europe: some key figures. Soc Psychiatry Psychiatr Epidemiol. (2021) 56:109–17. 10.1007/s00127-020-01909-6 32651594 PMC7847441

[16] Carabellese F Felthous AR. Closing Italian forensic psychiatry hospitals in favor of treating insanity acquittees in the community. Behav Sci Law. (2016) 34:444–59. 10.1002/bsl.2234 27256003

[17] Gualtieri G Traverso S Pozza A Ferretti F Carabellese F Gusinu R. Clinical risk management in High-Security Forensic Psychiatry Residences. Protecting patients and health professionals: perspectives and critical issues of the Law 81/2014. La Clinica Terapeutica. (2020) 171:e97–100. 10.7417/CT.2020.2196 32141478

[18] Ferracuti S Pucci D Trobia F Alessi MC Rapinesi C Kotzalidis GD. Evolution of forensic psychiatry in Italy over the past 40 years (1978–2018). Int J Law Psychiatry. (2019) 62:45–9. 10.1016/j.ijlp.2018.10.003 30616853

[19] Crocker AG Nicholls TL Seto MC Charette Y Côté G Caulet M. The national trajectory project of individuals found not criminally responsible on account of mental disorder in Canada. Part 2: the people behind the label. Can J Psychiatry. (2015) 60:106–16. 10.1177/070674371506000303 25886686 PMC4394710

[20] Hu J Yang M Huang X Coid J. Forensic psychiatry in China. Int J Law Psychiatry. (2011) 34:7–12. 10.1016/j.ijlp.2010.11.002 21159382

[21] Li G Gutheil TG Hu Z. Comparative study of forensic psychiatric system between China and America. Int J Law Psychiatry. (2016) 47:164–70. 10.1016/j.ijlp.2016.04.002 27292971

[22] Chen C Ou J-J Wang X-P. Guidelines on disposition of forensic psychiatric patients are urgent needed in China. J Forensic Leg Med. (2013) 20:823–4. 10.1016/j.jflm.2013.06.031 24112329

[23] Murasugi K Tsukahara T Washizuka S. The development and trial of a medication discontinuation program in the department of forensic psychiatry. Ann Gen Psychiatry. (2015) 14:11. 10.1186/s12991-015-0049-z 25788969 PMC4363327

[24] Haraguchi T Fujisaki M Shiina A Igarashi Y Okamura N Fukami G. Attitudes of Japanese psychiatrists toward forensic mental health as revealed by a national survey. Psychiatry Clin Neurosci. (2011) 65:150–7. 10.1111/j.1440-1819.2010.02180.x 21414090

[25] Malathesh BC Das S. Being a forensic psychiatrist in India: responsibilities, difficulties, and criticalities. Indian J Psychol Med. (2017) 39:732–6. 10.4103/IJPSYM.IJPSYM_334_17 29284802 PMC5733419

[26] Nambi S Ilango S Prabha L. Forensic psychiatry in India: past, present, and future. Indian J Psychiatry. (2016) 58:S175. 10.4103/0019-5545.196827 28216766 PMC5282612

[27] Hassan T Nizami AT Asmer MS. Forensic psychiatric service provision in Pakistan and its challenges. BJPsych Int. (2017) 14:40–4. 10.1192/S205647400000177X 29093938 PMC5618814

[28] Albarbari HS Al-Awami HM Bazroon AA Aldibil HH Alkhalifah SM Menezes RG. Criminal behavior and mental illness in the Arab world. J Forensic Sci. (2021) 66:2092–103. 10.1111/1556-4029.14882 34498734

[29] Every-Palmer S Brink J Chern TP Choi W-K Hern-Yee JG Green B. Review of psychiatric services to mentally disordered offenders around the Pacific Rim. Asia-Pacific Psychiatry. (2014) 6:1–17. 10.1111/appy.12109 24249353

[30] Eytan A Ngirababyeyi A Nkubili C Mahoro PN. Forensic psychiatry in Rwanda. Glob Health Action. (2018) 11:1509933. 10.1080/16549716.2018.1509933 30156144 PMC6116697

[31] Ogunlesi AO Ogunwale A Wet PD Roos L Kaliski S. Guest editorial: forensic psychiatry in Africa: prospects and challenges. Afr J Psychiatry. (2012) 15:3–7. 10.4314/ajpsy.v15i1.1 22344757

[32] Sukeri K Betancourt OA Emsley R Nagdee M Erlacher H. Forensic mental health services: current service provision and planning for a prison mental health service in the Eastern Cape. South Afri J Psychiatry. (2016) 22:5. 10.4102/sajpsychiatry.v22i1.787 30263155 PMC6138153

[33] O'Donahoo J Simmonds JG. Forensic patients and forensic mental health in victoria: legal context, clinical pathways, and practice challenges. Austr Soc Work. (2016) 69:169–80. 10.1080/0312407X.2015.1126750

[34] Ministry of Health NZ. Census of Forensic Mental Health Services 2005. (2007). Available online at: https://www.health.govt.nz/publication/census-forensic-mental-health-services-2005 (accessed March 26, 2022).

[35] Ogunlesi AO Ogunwale A. Correctional psychiatry in Nigeria: dynamics of mental healthcare in the most restrictive alternative. BJPsych Int. (2018) 15:35–8. 10.1192/bji.2017.13 29953117 PMC6020904

[36] Kirmayer LJ Rousseau C Lashley M. The place of culture in forensic psychiatry. J Am Acad Psychiatr Law Online. (2007) 35:98–102. 17389351

[37] Sharma S Sharma G. Exploring evolving concepts and challenges in forensic psychiatry. World Psychiatry. (2006) 5:97–8. 16946949 PMC1525120

[38] Velinov VT Marinov PM. Forensic psychiatric practice: worldwide similarities and differences. World Psychiatry. (2006) 5:98–9. 16946950 PMC1525133

[39] Bourget D Chaimowitz G. Forensic psychiatry in Canada: a journey on the road to specialty. J Am Acad Psychiatr Law Online. 2010) 38:158–62. 20542934

[40] Almanzar S Katz CL Harry B. Treatment of mentally ill offenders in nine developing Latin American Countries. J Am Acad Psychiatry Law. (2015) 43:340–9. 26438812

[41] Saberi SM Mirsepassi GR. Forensic psychiatry in Iran. Iran J Psychiatry Behav Sci. (2013) 7:1–3. PMC3939981 24644492

[42] Friedman SH. No worries, mate: a forensic psychiatry sabbatical in New Zealand. J Am Acad Psychiatry Law. (2013) 41:407–11. 24051594

[43] Fitch WL. Forensic Mental Health Services in the United States: 2014. Alexandria, VA: National Association of State Mental Health Program Directors (2014). Available online at: https://nasmhpd.org/content/forensic-mental-health-services-united-states-2014 (accessed March 26, 2022).

[44] Hare Duke L Furtado V Guo B Völlm BA. Long-stay in forensic-psychiatric care in the UK. Soc Psychiatry Psychiatr Epidemiol. (2018) 53:313–21. 10.1007/s00127-017-1473-y 29387921 PMC5842247

[45] Fovet T Thibaut F Parsons A Salize H-J Thomas P Lancelevée C. Mental health and the criminal justice system in France: a narrative review. Forensic Sci Int Mind Law. (2020) 1:100028. 10.1016/j.fsiml.2020.100028 35996435 PMC9387428

[46] Seppänen A Joelsson P Ahlgren-Rimpiläinen A Repo-Tiihonen E. Forensic psychiatry in Finland: an overview of past, present and future. Int J Ment Health Syst. (2020) 14:29. 10.1186/s13033-020-00362-x 32322299 PMC7164302

[47] Cid RD. Insane defendants and forensic convicts: before and after the onset of the new forensic psychiatry network and the criminal justice system reform in Chile. Curr Opin Psychiatry. (2010) 23:458–62. 10.1097/YCO.0b013e32833bb31a 20683181

[48] Asokan TV. Forensic psychiatry in India: the road ahead. Indian J Psychiatry. (2014) 56:121–7. 10.4103/0019-5545.130479 24891697 PMC4040057

[49] Völlm BA Clarke M Herrando VT Seppänen AO Gosek P Heitzman J. European Psychiatric Association (EPA) guidance on forensic psychiatry: evidence based assessment and treatment of mentally disordered offenders. Eur Psychiatry. (2018) 51:58–73. 10.1016/j.eurpsy.2017.12.007 29571072

[50] Salize HJ Dreßing H Kief C. Placement Treatment of Mentally Ill Offenders–Legislation Practice in EU Member States. Mannheim: Central Institute of Mental Health (2005). Available online at: https://ec.europa.eu/health/ph_projects/2002/promotion/fp_promotion_2002_frep_15_en.pdf (accessed March 26, 2022).

[51] Gunn J Mevis P. Adversarial versus inquisitorial systems of trial and investigation in criminal procedure. In: Goethals K, editor, Forensic Psychiatry and Psychology in Europe: A Cross-Border Study Guide. Cham: Springer International Publishing (2018). p. 3–17. 10.1007/978-3-319-74664-7_1

[52] Partlett W Ip EC. Is socialist law really dead. NYU J Int'l L Pol. (2015) 48:463–512.

[53] Quigley J. Socialist law and the civil law tradition. Am J Comp Law. (1989) 37:781–808. 10.2307/840224

[54] Oosterhuis H Loughnan A. Madness and crime: historical perspectives on forensic psychiatry. Int J Law Psychiatry. (2014) 37:1–16. 10.1016/j.ijlp.2013.09.004 24156902

[55] Koskinen L Likitalo H Aho J Vuorio O Meretoja R. The professional competence profile of Finnish nurses practising in a forensic setting. J Psychiatr Ment Health Nurs. (2014) 21:320–6. 10.1111/jpm.12093 23789940

[56] Nakatani Y. Challenges in interfacing between forensic and general mental health: a Japanese perspective. Int J Law Psychiatry. (2012) 35:406–11. 10.1016/j.ijlp.2012.09.021 23040709

[57] Scottish Government. Independent Forensic Mental Health Review: Final Report. (2021). Available online at: http://www.gov.scot/publications/independent-forensic-mental-health-review-final-report/ (accessed March 26, 2022).

[58] Razzouk D Caparroce DC Sousa A. Community-based mental health services in Brazil. Consorti Psychiatr. (2020) 1:60–70. 10.17650/2712-7672-2020-1-1-60-70 PMC11047271 38680388

[59] Xia L Jiang F Rakofsky J Zhang Y Shi Y Zhang K. Resources and workforce in top-tier psychiatric hospitals in China: a nationwide survey. Front Psychiatry. (2021) 12:573333. 10.3389/fpsyt.2021.573333 33716804 PMC7943845

[60] Krasnov V Gurovich I Bobrov A. Russian Federation: mental healthcare and reform. Int Psychiatry. (2010) 7:39–41. 10.1192/S1749367600005725 31508031 PMC6734957

[61] World Health Organization. WHO-AIMS Report on Mental Health System in Pakistan. 2009. WHO Office: World Health Organization (2018). Available online at: https://www.mindbank.info (accessed March 26, 2022).

[62] World Health Organization. WHO-AIMS Report on Mental Health System in South Africa. Cape Town: WHO and Department of Psychiatry and Mental Health, University of Cape Town (2007). Available online at: https://www.mindbank.info (accessed March 26, 2022).

[63] World Health Organization. WHO-AIMS Report on Mental Health System in Nigeria. Nigeria: Ministry of Health (2006). Available online at: https://www.mindbank.info (accessed March 26, 2022).

[64] World Health Organization. WHO-AIMS Report on Mental Health System in Egypt. Cairo: WHO and Ministry of Health Retrieved November (2006). Available online at: https://www.mindbank.info (accessed March 26, 2022).

[65] World Health Organization. WHO-AIMS Report on Mental Health System in the Islamic Republic of Iran. Tehrran: World Health Organization—Assessment Instrument for Mental Health Systems (2006). Available online at: https://www.mindbank.info (accessed March 26, 2022).

[66] Ministerio de Salud Chile. Plan nacional de salud mental 2017-2025. (2017) Available online at: https://www.minsal.cl (accessed March 26, 2022).

[67] Australian Institute of Health Welfare. Mental Health Services in Australia. Canberra, ACT: Australian Institute of Health and Welfare (2022) Available online at: https://www.aihw.gov.au/reports/mental-health-services/mental-health-services-in-australia (accessed March 26, 2022).

[68] Livingston J. A statistical survey of canadian forensic mental health inpatient programs. Healthcare Quarterly. (2006) 9:56–61. 10.12927/hcq..18104 16640134

[69] Numbers of psychiatric beds for OECD countries were retrieved from: www.stats.oecd.org

[70] Di Lorito C Castelletti L Lega I Gualco B Scarpa F V?llm B. The closing of forensic psychiatric hospitals in Italy: determinants, current status and future perspectives. A scoping review. Int J Law Psychiatry. (2017) 55:54–63. 10.1016/j.ijlp.2017.10.004 29157512

