# Corrigendum: Impact of legal traditions on forensic mental health treatment worldwide

**DOI:** 10.3389/fpsyt.2022.1057552

**Published:** 2022-10-27

**Authors:** Pavlos Beis, Marc Graf, Henning Hachtel

**Affiliations:** Department of Forensics, University Psychiatric Clinic Basel, Basel, Switzerland

**Keywords:** forensic psychiatry, mental health, forensic service, legal frameworks, treatment standards

In the published article, part of the text was not inserted.

A correction has been made to the **Abstract**, sub-section “Results.” This sentence previously stated:

**Results:** Forensic treatment admission varies significantly around the world. There are countries that do not recognize forensic psychiatry as a subspecialty, whereas other countries apply insufficient forensic training. Most countries provide inpatient treatment for mentally ill offenders. However, service organization varies, including where the services are delivered (prisons, high-security hospitals, and general psychiatric departments). Forensic services are mainly centralized, although the need for outpatient care is emerging.

The corrected sentence appears below:

**Results:** Forensic treatment admission varies significantly around the world. There are countries that do not recognize forensic psychiatry as a subspecialty, whereas other countries apply insufficient forensic training. Most countries provide inpatient treatment for mentally ill offenders. However, service organization varies, including where the services are delivered (prisons, high-security hospitals, and general psychiatric departments). Forensic services are mainly centralized, although the need for outpatient care is emerging. This manuscript updates the findings of a chapter by Anne G. Crocker, James D. Livingston, and Marichelle C. Leclair that conducted an international review on the organization of forensic mental health services internationally, by legal framework. We were also inspired by the classification of legal frameworks from that chapter conducting the present review. Building upon that chapter we reviewed current literature about forensic mental health treatment from countries with different legal traditions, accentuated similarities and differences among them and highlighted that further follow-up research is needed, aiming the optimization of forensic treatment standards.

In the published article, the reference for citation (6) was incorrectly written as “Roesch R, Cook AN. *Handbook of Forensic Mental Health Services*. New York, NY: Routledge (2017). doi.org/10.4324/9781315627823. It should be “Crocker AG, Livingston JD, Leclair MC. Forensic mental health systems internationally. In: Roesch R, Cook AN, editors, *Handbook of Forensic Mental Health Services*. New York, NY: Routledge (2017). p. 3–76.”

The authors apologize for these errors and state that this does not change the scientific conclusions of the article in any way. The original article has been updated.

## Publisher's note

All claims expressed in this article are solely those of the authors and do not necessarily represent those of their affiliated organizations, or those of the publisher, the editors and the reviewers. Any product that may be evaluated in this article, or claim that may be made by its manufacturer, is not guaranteed or endorsed by the publisher.

